# Spatial integration of dendrites in fast-spiking basket cells

**DOI:** 10.3389/fnins.2023.1132980

**Published:** 2023-04-04

**Authors:** Ming Liu, Xiaojuan Sun

**Affiliations:** School of Science, Beijing University of Posts and Telecommunications, Beijing, China

**Keywords:** fast-spiking basket cells, compartmental model, dendritic non-linearity, spatial integration, hippocampus

## Abstract

Dendrites of fast-spiking basket cells (FS BCs) impact neural circuit functions in brain with both supralinear and sublinear integration strategies. Diverse spatial synaptic inputs and active properties of dendrites lead to distinct neuronal firing patterns. How the FS BCs with this bi-modal dendritic integration respond to different spatial dispersion of synaptic inputs remains unclear. In this study, we construct a multi-compartmental model of FS BC and analyze neuronal firings following simulated synaptic protocols from fully clustered to fully dispersed. Under these stimulation protocols, we find that supralinear dendrites dominate somatic firing of FS BC, while the preference for dispersing is due to sublinear dendrites. Moreover, we find that dendritic diameter and Ca^2+^-permeable AMPA conductance play an important role in it, while A-type K^+^ channel and NMDA conductance have little effect. The obtained results may give some implications for understanding dendritic computation.

## 1. Introduction

Across all cortical circuits, GABAergic interneurons represent a minority yet serve a critical impact on modulating circuit functions (Hu et al., 2014). They set a limited time window for synaptic integration and plasticity in principal neurons and coordinate synchronous activity during neuronal oscillations (Buzsaki and Draguhn, 2004; Hu et al., 2010; Chiovini et al., 2014). As one particular type of GABAergic interneuron, fast-spiking basket cells (FS BCs) can be distinguished by their morphological properties, expression of molecular markers, and functional characteristics (Hu et al., 2014). For fast-spiking basket cells, their action potentials present short duration and fast-spiking phenotype (Jonas et al., 2004), and synapses located on their dendrites contain GluR2-lacking Ca^2+^-permeable AMPA (cp-AMPA) receptors and low levels of NMDA receptors (Geiger et al., 1997; Adesnik and Nicoll, 2007; Freund and Katona, 2007; Povysheva et al., 2013). Among them, cp-AMPARs possess faster deactivation kinetics (Geiger et al., 1995; Isaac et al., 2007), and FS BCs with cp-AMPA conductances in rat prefrontal cortex are reported to exhibit prominent synaptic facilitation (Wang and Gao, 2010). These features above make FS BCs essential regulators of network oscillations (Jiang et al., 2016) and memory consolidation (Xia et al., 2017). Due to these essential roles that FS BCs played in the brain, investigating the information processing mechanisms in them is vital for understanding the correlated brain functions.

Dendritic integration, a fundamental part of neuronal information processing, is classified into linear, sublinear, and supralinear integration. Previous studies have revealed that dendrites of interneurons exhibit mostly linear or sublinear integration (Tzilivaki et al., 2022), and dendrites of some CA1 interneurons could show supralinear integration (Katona et al., 2011). Except for these neurons, dendritic integration has also been discussed in other neurons, such as granule cells (Krueppel et al., 2011), etc. Neuronal response to spatiotemporal synaptic inputs depends heavily on the type of dendritic integration. In CA1 pyramidal neurons with supralinear integration, stimulations on synapses clustered within a branch can cause more robust responses than on dispersed allocated synapses (Poirazi et al., 2003). While for other neuron types with sublinear dendrites, their response is usually sensitive to scattered synaptic inputs (Cazé et al., 2013). Meanwhile, the dendritic integration mode can affect the output precision (Gasparini and Magee, 2006) and further neural oscillation (Wang, 2010).

Dendritic integration has been found to be related to neuronal physiological properties (Araya et al., 2006; Vervaeke et al., 2012; Kamijo et al., 2014; Mueller and Egger, 2020; Di Maio, 2021). For example, narrow dendritic diameters produce more significant local input impedance, thus inducing prominent sublinear integration in stellate cells (Abrahamsson et al., 2012). Sodium ion channels and NMDA receptors are the main amplification mechanism for supralinear integration of pyramidal neurons (Nevian et al., 2007). Alike, dendritic properties such as fast inactivating K^+^ current (Shibata et al., 2000) and dendritic morphology (Hartveit et al., 2022), or synaptic conductances can affect spatial integration and the first spike latency, which together provide precise mediation of the postsynaptic neurons.

Previous studies on spatial integration mainly focused on fully clustered (Carter et al., 2007; Gökçe et al., 2016; Dembrow and Spain, 2022) or fully dispersed (Farinella et al., 2014) synaptic inputs, without paying attention to the intermediate state, which is difficult to realize in the experiment, thus hindering understanding of the specific connection mode in the brain. Recently, supralinear and sublinear integration coexisting(bi-modal integration) on the same dendrite of FS BCs has been reported (Tzilivaki et al., 2019). They reveal that FS BCs present dispersed preference due to the specific morphological features, A-type potassium channels, and the existence of sublinear dendrites. Nevertheless, the underlying mechanisms in FS BCs to transform discriminate spatial synaptic inputs and the role of the two non-linear dendrites in it remain elusive.

In this paper, we aim to investigate how supralinear and sublinear dendrites of FS BCs influence their responses to synaptic inputs with different spatial dispersion. Using a biophysically-detailed compartmental model of a CA3 fast-spiking basket cell, under the knowledge of sublinear dendrites making FS BCs prefer dispersed spatial synaptic inputs as reported by Tzilivaki et al. (2019), we further find that supralinear dendrites play a dominant role in FS BCs' firing response. Responses of FS BCs to spatial synaptic inputs stimulus of the whole dendritic tree are consistent with that to the supralinear dendrites stimulus only. And this result is independent of the proportion of sublinear dendrites to supralinear dendrites. To understand the mechanisms of this spatial integration, we alter several biophysical properties in dendrites. We find that dendritic diameter varies somatic response in a nonmonotonic way and is the determining factor in regulating the precise firing of FS BCs. Then, we apply the same spatial simulation protocols after blocking cp-AMPA and NMDA synapses separately. The results demonstrate that cp-AMPA receptors improve the integration capacity immensely, while the presence of NMDA currents is insufficient for active dendritic spikes. Our findings may provide insights into the role of spatial integration in interneurons, leading to the speed and temporal precision operation of GABA release and the regulation of interneurons in memory updating further.

## 2. Materials and methods

### 2.1. Compartmental modeling

Compartmental model of the CA3 fast-spiking basket cell is implemented and run within the NEURON simulation environment (Hines and Carnevale, 1997). The detailed dendritic morphology model J31a.CNG.swc is obtained from the NeuroMorpho.org database uploaded by Tukker et al. (2013), consisting of 217 compartments. Dendrites distance from soma less than 100 μm is defined as proximal dendrites, otherwise as distal dendrites (Hu et al., 2014). Axonal compartments of B13a.CNG.swc in the same brain area is adopted since the lack of axons in the J31a model. And segment number of all compartments is set the same as the number of synaptic inputs.

Passive cable properties and active conductance of ionic channels in the model are all based on experimental data (Supplementary Tables 1, 2) (Goldberg et al., 2003; Hu et al., 2010; Konstantoudaki et al., 2014). Fast Na^+^ ion channels (Hu et al., 2014) and delayed rectifier K^+^ current are inserted into all compartments. Slow inactivation K^+^, two Ca^2^^+^-dependent potassium, and h currents are inserted only in soma. Other channels such as A-type K^+^ are for proximal and distal dendrites, while L-, N-, and T-type Ca^2^^+^ currents are added to each dendritic and somatic compartment. Meanwhile, all compartments include a calcium buffering mechanism except axon (Konstantoudaki et al., 2014). For synaptic inputs, synapses with Ca^2^^+^ permeable AMPA (cp-AMPA) and NMDA receptors are considered, and inhibitory synapses are not considered in our simulation due to the very low ratio of them in all incoming inputs (Freund and Katona, 2007; Hu et al., 2014). Values of all synaptic weights are consistent with Tzilivaki et al. (2019).

### 2.2. Spatial simulation protocols

In all simulation protocols, 20 pairs of cp-AMPA and NMDA synapses are randomly located on the dendrites with an average diameter being less than 1.2 μm are activated by a 50 Hz Poisson spike train (Emri et al., 2001). In the following, if not specialized, all the dendrites refer to those with diameters less than 1.2 μm. In order to study the different roles of supralinear and sublinear dendrites on the response of FS BCs, synaptic stimulations are put into the whole dendrites, only supralinear dendrites or sublinear dendrites, respectively. And the locations of the activated synapses change from fully clustered on a single dendritic branch to fully dispersed on the whole dendritic tree, among which the selected compartment for dispersing is random. The results obtained in this paper are the average over *N* since simulation protocols repeat *N* times. Here, *N* is the number of non-linear dendrites when the activated synapses are located on the whole dendrites, and is the number of supralinear/sublinear dendrites when the activated synapses are located only on supralinear/sublinear dendrites.

## 3. Results

### 3.1. Preference of FS BCs to dispersed spatial synaptic inputs

**Figure 1:**
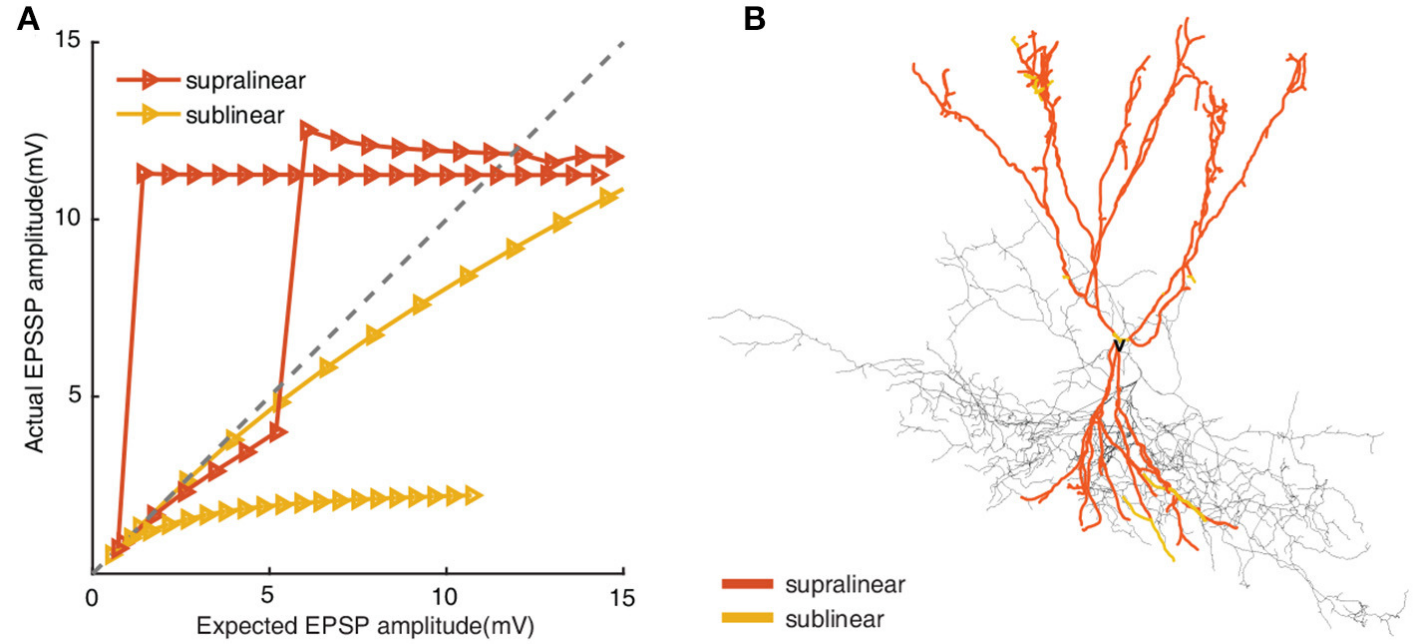
**(A)** Input-output relationships from supralinear and sublinear dendrites in FS BC model. Increasing numbers of synapses(from 1 to 20 pairs) activate on each compartment. The *x*-axis presents the linear summed EPSP, while the *y*-axis shows the actual EPSP amplitude recorded in the soma. The gray line indicates linear summation. **(B)** Morphological reconstructions of the modeled FS BC with supralinear (red) and sublinear (yellow) dendrites. The removed dendritic branches are also colored according to the actual integrated properties in each compartment, but the removed dendritic branches are not considered in subsequent simulations.

Unlike the former opinion that dendrites integrate signals linearly, fast-spiking basket cells have been authenticated to perform both supralinear and sublinear computations. To investigate the different roles of supralinear and sublinear dendrites on the response of FS BCs, we need to identify each dendrite of FS BCs to be supralinear or sublinear. Toward this goal, we stimulate gradually increasing cp-AMPA and NMDA synaptic inputs(from 1 to 20 pairs) to each dendrite and block sodium channels in soma and axon to avoid backpropagation effects. By comparing the actual EPSP (measured EPSP) recorded in soma with linearly summed EPSP (expected EPSP), dendritic compartments are labeled as supralinear/sublinear if measured EPSP is larger/less than the expected EPSP, as shown in Figure 1. And we find that the studied FS BC has 151 supralinear dendrites and 17 sublinear ones.

**Figure 2:**
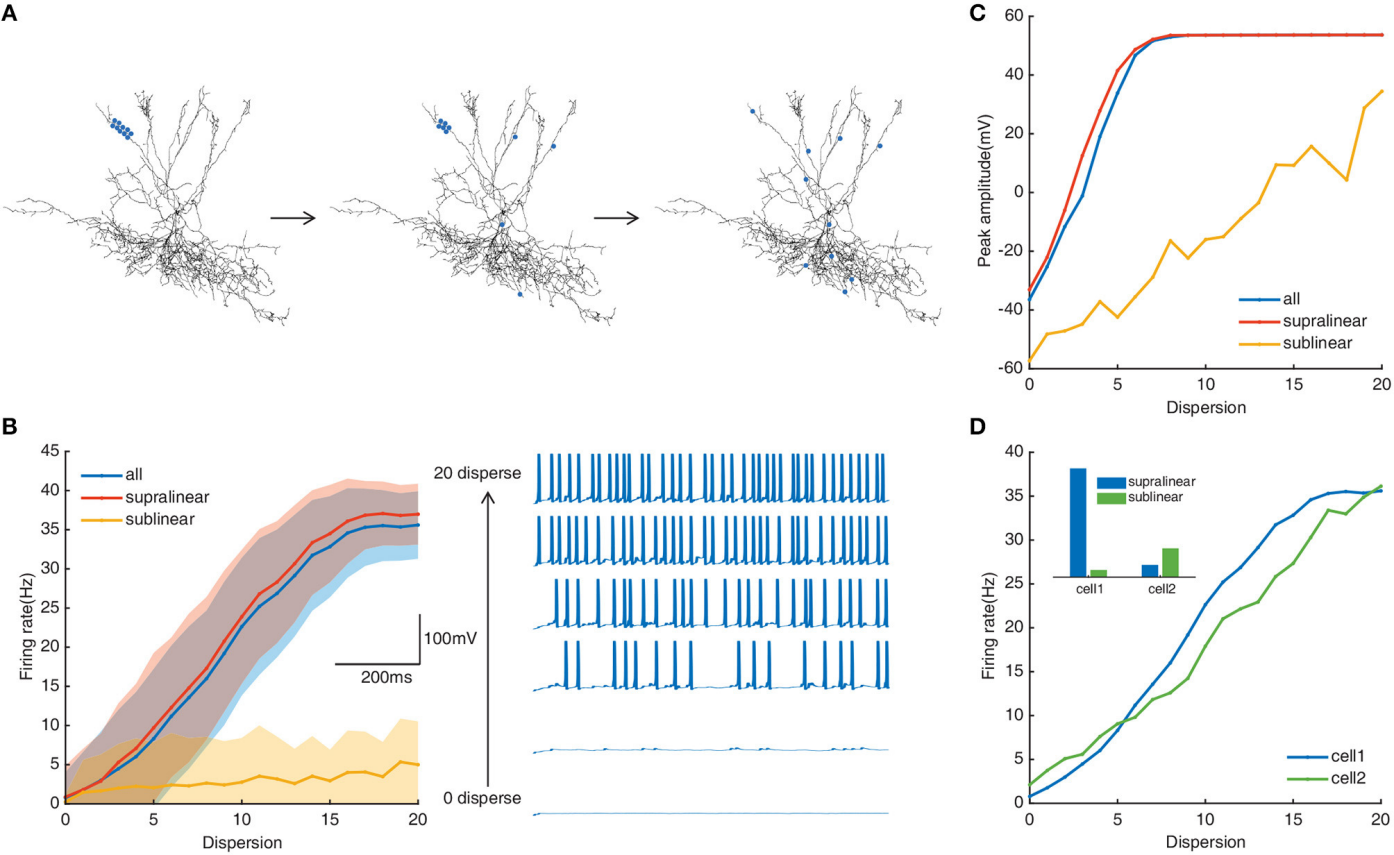
**(A)** Schematic diagram of spatial simulation protocols. Twenty pairs of synapses randomly located on selected dendrites from fully clustered to fully dispersed (blue dots represent the stimulation site). All processes cycle for *N* times (*N* is the number of stimulated branches). Output is recorded in the soma. **(B)** Left: Traces of the somatic firing rate in the whole dendrites (blue line), supralinear dendrites (red line), and sublinear dendrites (yellow line) under the spatial simulation protocol. Every shaded area denotes the corresponding standard deviation. Right: Example of somatic membrane potentials on a selected dendrite (every three dispersion degrees). **(C)** Peak membrane potential record in the whole dendritic tree, supralinear dendrites, and sublinear dendrites. **(D)** Somatic firing rate of two neuron models. Cell1 represents the neuron with a ratio of supralinear dendrites to sublinear dendrites of 8.88 (blue); cell2 represents the neuron with a ratio of 0.25 (green). Inset denotes the histogram of the number of non-linear dendrites in two neurons (blue: supralinear, green: sublinear).

Dendritic integration plays important role in neuronal responses to different spatial synaptic inputs (Tran-Van-Minh et al., 2015). We implement diverse spatial synaptic input patterns on selected dendrites to investigate the roles of different non-linear dendrites in FS BCs' responses (see “Methods”). As shown in Figure 2A, synaptic inputs are placed randomly on dendrites from fully clustered to fully dispersed. Responses of FS BC to synaptic inputs subjected to the whole dendrites, to only supralinear or sublinear dendrites are exhibited by blue, red, and orange lines in Figures 2B, C, respectively. The firing rate of the whole dendritic tree increased from an average of 0.79 ± 3.2Hz to a maximum of 35.6 ± 4.3Hz. In Figures 2B, C, both somatic firing rate and peak amplitude of FS BC's spike train increase with synaptic inputs' dispersity when active synapses are located at the whole dendrites or only on supralinear dendrites. For active synapses located at only sublinear dendrites, the somatic firing rate doesn't change too much, and peak amplitude increases while can't reach the peak value as supralinear dendrites. It indicates that supralinear dendrites prefer dispersed synaptic inputs with the existence of sublinear ones, and they play a dominant role since the trajectories are similar for synaptic inputs putting only on supralinear dendrites and the whole dendrites. With the variation of peak amplitude, it can be seen that dendritic spikes occurring on supralinear dendrites make the membrane potential of soma come to the peak (Goldberg et al., 2003), not sublinear ones.

The ratio of supralinear dendrites over the number of sublinear dendrites of our model is about 8.88. To test whether this cluster-disperse response is related to the ratio of the non-linear dendrites, we repeat the same simulation protocols on another FS basket cell with more sublinear dendrites than supralinear dendrites (ratio = 0.25). Results show that although the ratio is diametrically changed, this cell also prefers dispersed synaptic inputs (Figure 2D), and supralinear dendrites play a dominant role in this preference too. It indicates that FS BCs' preference for dispersed synaptic inputs with the existence of sublinear ones is independent of the nonl-inear dendrites' ratio. This independence property may provide a way for us to deduce the spatial dispersion of synaptic inputs from the firing rate of FS BCs.

In summary, under simulated synaptic protocols from fully clustered to fully dispersed, we find that supralinear dendrites play a dominant role in the somatic firings of FS BCs. They can trigger and transmit enough dendritic spikes to the soma. Meanwhile, the preference of FS BCs for dispersed synaptic inputs is due to the existence of sublinear dendrites.

### 3.2. Dendritic diameters play a crucial role in the spatial responses of FS BCs

**Figure 3:**
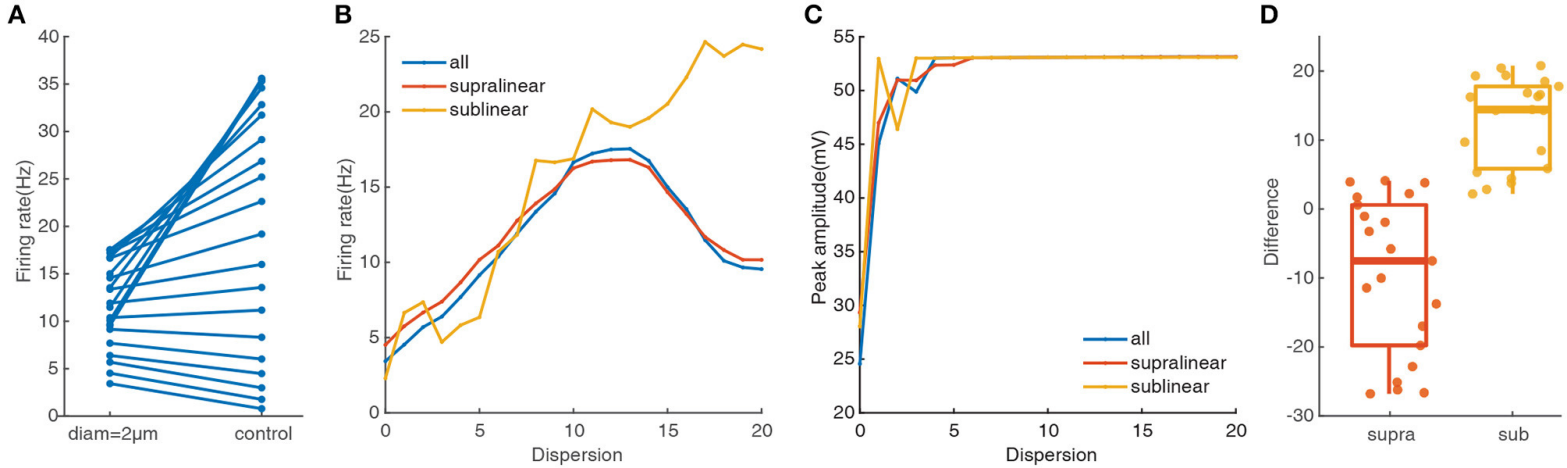
Dendritic morphology affect notably on the cluster-disperse spatial responses. **(A)** Firing rate evoked in the neuron after and before changing the diameter. The points located at the right side of the figure are the somatic firing rate under the normal condition when the dendritic diameters have not changed. All the following parts are the same. **(B)** Traces of firing rate in the whole dendrites and different non-linear dendrites after changing the diameter. **(C)** Peak membrane potential record in the whole dendrites and other non-linear dendrites under 2 μm diameter. **(D)** Box plot and scatter plot of the differences in firing rate between the normal condition and changing the diameter in two non-linear dendrites. Red: supralinear dendrites, yellow: sublinear dendrites.

Dendritic morphology and A-type potassium channels have been reported to contribute to the dispersed preference in FS BCs (Hu et al., 2014). To explore their impact on responses of FS BCs under spatial synaptic inputs, we repeat the above simulation protocols after increasing the dendritic diameter to 2 μm. As shown in Figure 3A, compared with the data on the right side that dendritic diameters are unchanged, the somatic firing rate with spatial synaptic inputs located on the whole dendrites tends to concentrate to lower values, ranging from 3.4 Hz to 17.54 Hz. And its nonmonotonic variation tendency is still consistent with the one under stimulations only on the supralinear dendrites, even though somatic firing rate increases with a dispersion of spatial synaptic inputs put only on sublinear dendrites (Figure 3B). This diversity is also reflected in peak amplitude. As shown in Figure 3C, whether synapses are activated in the whole dendrites or supralinear/sublinear dendrites, the membrane potential of soma can always reach the peak and the difference becomes smaller than the results shown in Figure 2C. It also reveals that the narrow diameter of dendrites may hamper the response produced by clustered inputs, while a large diameter may reduce the response produced by dispersed inputs.

To more intuitively reflect the changing response in different non-linear dendrites, we use Student's t-test for statistically compared. Variations between the two non-linear dendrites are significant(*p* = 0.00011), where the median in the sublinear dendrites is larger than 10 (Figure 3D). Together these results further confirm that supralinear dendrites dominate the firing in FS BCs, and dendritic diameter plays a vital role in the spatial response of FS BCs which is also an essential factor in determining the firing properties of sublinear dendrites.

**Figure 4:**
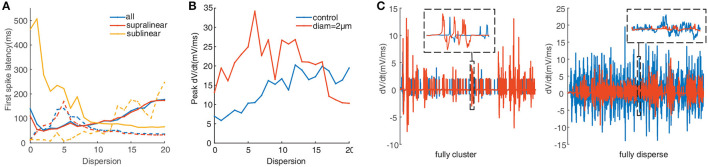
**(A)** First spike latency before (dotted lines) and after (solid lines) changing the diameter. **(B)** Peak dV/dt against dispersion of synaptic inputs calculated before (blue) and after (red) changing the diameter. **(C)** Detailed differentiated membrane potential (dV/dt) traces under fully clustered and fully dispersed, insets are the enlarged image. The line color is consistent with the legend color in **(B)**.

To further test the impacts of dendritic morphology on EPSP-spike coupling, we analyze the first spike latency for synaptic inputs located on the whole, the supralinear dendrites, and sublinear dendrites respectively. First spike latency is defined as the interval between the onset of the simulation and the peak of the first spike. Under control conditions (diameter of dendrites doesn't change), the dependence of first spike latency on dispersion for spatial synaptic inputs on the whole dendrites is still consistent with the ones on the supralinear dendrites. And the nonmonotonic dependence of first spike latency on dispersion (Figure 4A, blue and red dotted line) is different from the increasing one for inputs on only sublinear dendrites (Figure 4A, yellow dotted line). When the dendritic diameter changes to 2 μm, the dependence of first spike latency has changed conversely. For example, first spike latency decreases with dispersion (Figure 4A, yellow solid line), which is contrary to the one under control condition. And for the other two cases, first spike latency decreases first and then increases with dispersion (Figure 4A, blue and red solid line), which is also different from the ones increasing first and then decreasing for the control condition (Figure 4A, blue and red dotted line). These obtained results indicate that dendritic diameter could alter signal transfer efficiency in FS BCs.

Peak of the derivation membrane potential(dV/dt) versus degree of dispersion can provide another perspective of investigation (Losonczy and Magee, 2006). The blue line in Figure 4B shows that the peak dV/dt rises gradually under control conditions. After increasing the dendritic diameter to 2 μm, the peak dV/dt is more eminent at lower dispersion, and then it clips to a lower value (Figure 4B, red line). Figure 4C presents the variation of dV/dt for fully clustered or dispersed stimulations. For fully clustered synaptic stimulation, dV/dt with the diameter being 2 μm fluctuates much more greatly than the one under control condition (Figure 4C, left), while vice versa for fully dispersed synaptic stimulation (Figure 4C, right). These results indicate that dendritic diameter is associated with the peak dV/dt, notably for higher dispersion stimulation.

**Figure 5:**
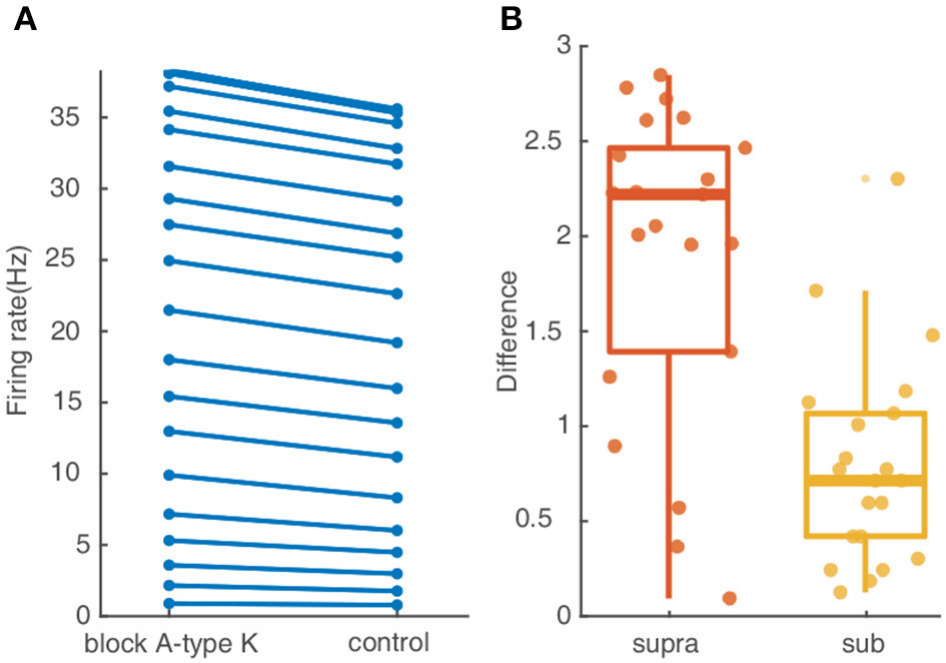
A-type K^+^ channel affect little on the cluster-disperse spatial responses. **(A)** Firing rate evoked in the neuron after and before blocking A-type K^+^ channels. **(B)** Box plot and scatter plot of the differences in firing rate between the normal condition and blocking the A-type K^+^ channel in two non-linear dendrites. Red: supralinear dendrites, yellow: sublinear dendrites.

A-type K^+^ channels are reported to be an important factor for controlling neuronal response. To investigate their contribution to the response of the studied FS BC to spatial synaptic inputs with different dispersions, we repeat the simulation protocols after setting the conductance of A-type K^+^ channels to zero. As shown in Figure 5A, the somatic firing rate for inputs on the whole dendrites increases slightly after blocking the A-type K^+^ channels. The difference in Figure 5B reflects somatic firing rises more in the supralinear dendrites than sublinear dendrites. Therefore A-type K^+^ channels contribute little effect on the cluster-disperse response.

In summary, these simulations demonstrate that the specific morphology features of FS BCs conduct the discrepancy in the cluster-disperse integration of different non-linear dendrites and play a key element in controlling the release timing precisely, while A-type K^+^ channels present minor effects.

### 3.3. Synaptic conductances determine responses and output precision of FS BCs to spatial synaptic inputs

**Figure 6:**
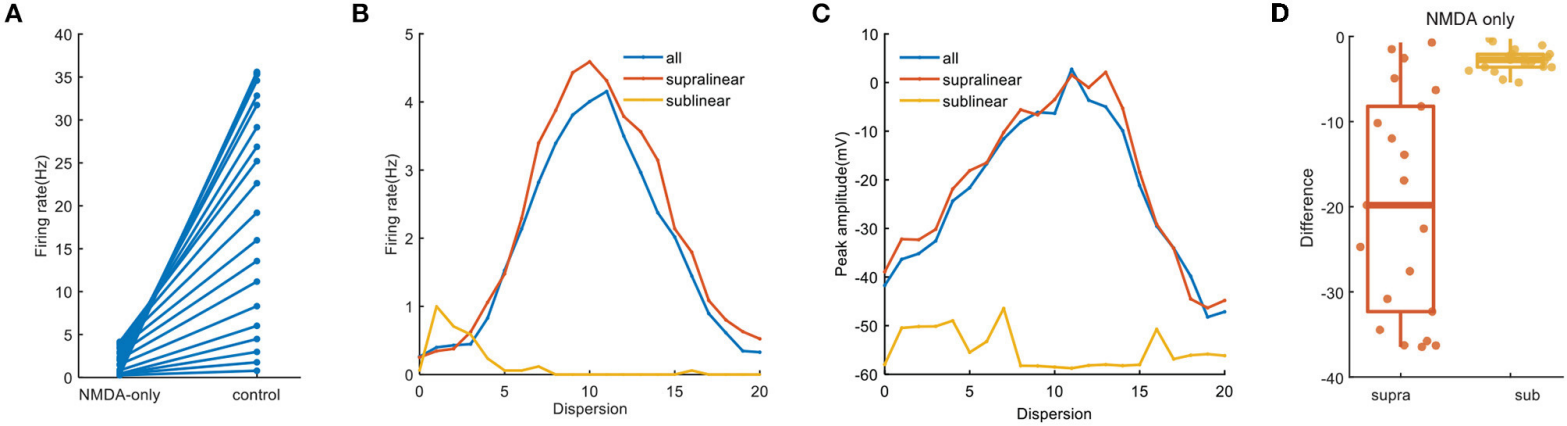
NMDA-only synapses on spatial responses. **(A)** Plot of firing rate in the whole dendrites for synaptic inputs having NMDA-only (same as the blue line in **B**) and normal condition. **(B)** Traces of firing rate with dispersion in the whole dendrites and two non-linear dendrites with NMDA synapses only. **(C)** Peak membrane potential with NMDA synapses only. **(D)** Box plot with scatter plot of the differences in firing rate between the normal condition and blocking cp-AMPA synapses in two non-linear dendrites.

Since the type of excitatory synapses influences synaptic integration, we reason that they may also act to the cluster-disperse spatial responses of FS BC (Schiller et al., 2000). To test this, we apply synapses consisting of only cp-AMPA or NMDA under the same stimulation protocols. For simulations involving NMDA-only, the neuron barely generates action potentials and the somatic firing rate range from 0.26 ± 1.2 Hz to 4.15 ± 5.6 Hz, which decreases from beta wave to alpha wave (Figure 6A). Same as Figure 3B, the disperse-sensitive response disappears. While the tendency is still increased first and then decreased, the difference between the two ends becomes smaller (Figure 6B). Noticed when synapses are added to the sublinear dendrites only, the somatic firing rate does not increase with the dispersion, this suggests that synaptic conductance does not affect the characteristics of sublinear dendrites. For peak amplitude, its maximum decreases to nearly zero (Figure 6C) as compared to the one under the control condition as exhibited in Figure 2C. Variation of dependence of peak amplitude on stimulation dispersion could explain why the somatic firing rate drops to alpha bands. The difference shown in Figure 6D reveals when synapses contain NMDA only, dendritic spikes on the supralinear dendrites reduce a lot, which indicates the necessity of cp-AMPA conductances for dendritic spikes.

**Figure 7:**
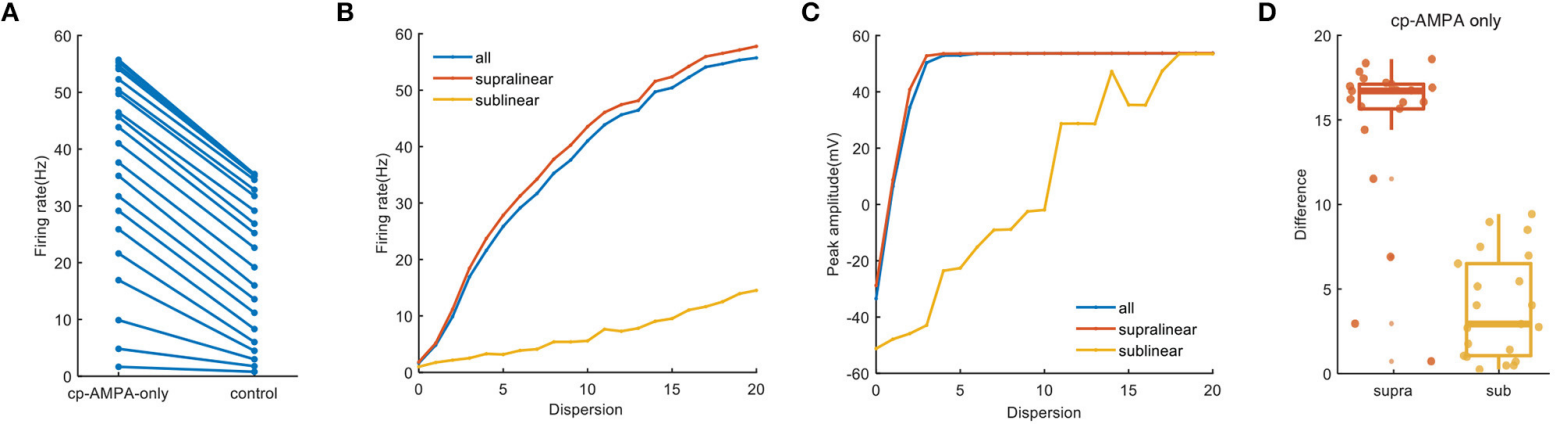
Cp-AMPA-only synapses on spatial responses. **(A)** Plot of firing rate in the whole dendrites for synaptic inputs having cp-AMPA only and normal condition. **(B)** Traces of firing rate with dispersion in the whole dendrites and two non-linear dendrites with cp-AMPA synapses only. **(C)** Peak membrane potential record in the whole dendrites and different non-linear dendrites with cp-AMPA synapses only. **(D)** Box plot with scatter plot of the differences in firing rate between the normal condition and blocking NMDA synapses in two non-linear dendrites. There are several outliers in the difference of supralinear dendrites.

When synapses contain cp-AMPA only, somatic firing in the whole dendrites increases significantly, from 1.65 Hz of minimum to 55.7 Hz, which is consistent with the supralinear and sublinear dendrites (Figures 7A, B). Meanwhile, the variation of peak amplitude with dispersion reveals the dispersion needed for reaching the maximum value of peak amplitude is lower (Figure 7C). In addition to several outliers at low dispersion, Figure 7D further presents a strong and uniform influence of cp-AMPA on the supralinear dendrites rather than the sublinear dendrites. These results demonstrate that cp-AMPA conductance is required for the dispersion-sensitive properties of FS BCs, which point out that it becomes another crucial element in spatial integration by inducing dendritic spikes.

**Figure 8:**
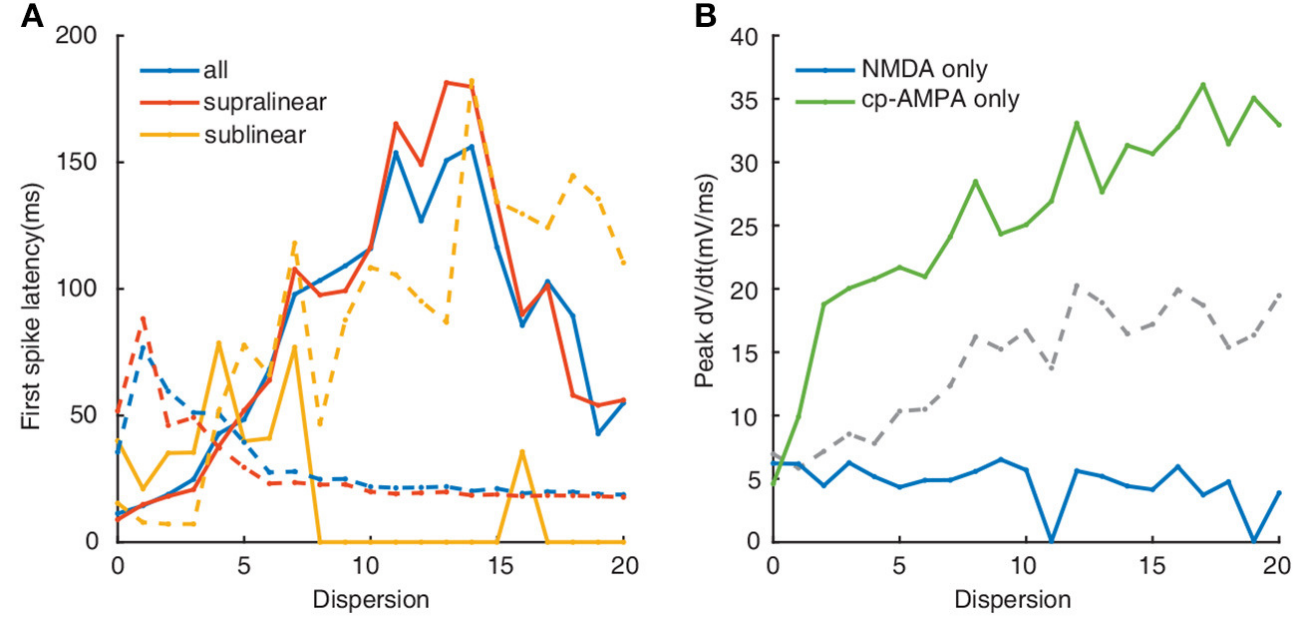
**(A)** First spike latency when applied NMDA synapses only (solid lines) and cp-AMPA synapses only(dotted line). Colors indicate as in legend. **(B)** Plot of peak dV/dt against dispersion of synaptic inputs. Colors (blue, green, and gray) indicate NMDA, cp-AMPA, and both cp-AMPA and NMDA synaptic synapses, respectively.

Synaptic conductances also affect the temporal input-output relationship of neurons (Wang and Liu, 2010; Di Maio et al., 2021). As illustrated in Figure 8, compared with the dotted lines in Figure 4A, the change of nonmonotonic trajectory in both cases is the same as that under normal conditions, which increases first and then decreases. When NMDA conductance is added only, the latency of the first spike is longer, and the existence of 0 in sublinear dendrites is due to the absence of firing (Figure 8A, solid yellow line). However, no matter how the first spike latency changes under different conditions, sublinear dendrites still react contrastively to others.

The calculation of peak dV/dt increases following the blockade of NMDA receptors, corresponding with the change of somatic outputs (Figure 8B, green line). In contrast, for NMDA-only conductance, the peak dV/dt decreases with the degree of dispersion (Figure 8B, blue line). Altogether these data indicate that cp-AMPA synapses can further reduce the transmission threshold of the dendritic spike, while the threshold increases sharply with NMDA inputs only.

In summary, we find that the faster kinetics of cp-AMPA synapses influences the response of spatial dispersion inputs in FS BCs. They can regulate both the summation and timing of dendritic spikes and further affect neuronal firing. On the other hand, NMDA conductance has a minor effect on this cluster-disperse response, and more effects may have to be studied on a longer time scale.

## 4. Discussion

Dendritic integration is one of the critical components of neuronal computation, and different type of integration leads to diverse spatial response (Kastellakis et al., 2015; Li and Gulledge, 2021). Exciting new findings show that the dendrites of fast-spiking basket cells in the hippocampus present both supralinear and sublinear integration (Tzilivaki et al., 2019) (Figure 1). How this bi-modal dendritic integration responds to synaptic inputs from fully clustered to fully dispersed of have not been previously examined. Here, using a compartmental model, we investigate how the supralinear and sublinear dendrites in FS BCs participate in the process of transforming the cluster-disperse patterns of synaptic inputs into outputs.

Our results demonstrate that supralinear dendrites play a dominant role in FS BCs' response to the cluster-disperse synaptic inputs, they dominate the somatic firing by generating dendritic spikes, and sublinear branches compute the disperse preference (Figure 2). Neurons employ a variety of mechanisms to combat spatial variability in synaptic inputs, and dendritic properties can significantly affect the ability of synaptic input to generate, propagate, and time action potentials (Psarrou et al., 2014). Similar to previous studies in other neurons, we find that dendritic morphology is the main cause of this diversity of spatial integration in non-linear dendrites (Single and Borst, 1998; Ran et al., 2020). Larger diameters diminish the disperse-sensitive responses in a nonmonotonic way of the whole and supralinear dendrites, while intensely increasing the firing in the sublinear dendrites (Figure 3). As the dendritic diameter is a morphological parameter of the neuron itself, the firing properties in sublinear dendrites completely change when the dendritic diameter alters to an abnormal value. Besides the firing rate, precise temporal propagation is essential in signal transfer, time course of synapses defines the time window for the firing of basket cells and their influence on the pyramidal neurons (London et al., 2002; Molineux et al., 2005; Wlodarczyk et al., 2013). The relationship between the first-spike latency and morphology shows an intriguing result on non-linear dendrites, supporting that this particular morphology of FS BCs determines its accurate timing transmission of information (Figure 4). Unlike previous studies, although K^+^ channels influence neuronal responses (Misonou et al., 2005; Tzilivaki et al., 2019), blocking the A-type K^+^ channels in the model has a minor influence on the cluster-disperse responses (Figure 5). Still, specificity in this model can not be ruled out.

The gating properties of cp-AMPA or NMDA receptors can also affect the participation of different non-linear dendrites in spatial integration (Mcbain and Dingledine, 1993; Isaac et al., 2007). Among them, cp-AMPARs mediate excitatory postsynaptic current raises and decays rapidly (Carter and Regehr, 2002; Walker et al., 2002), the EPSP arriving at the soma has a relatively short half-duration (Angulo et al., 1999; Jonas et al., 2004). Separately blocking the synaptic conductance in the model, we observe that activating NMDA synapses only causes minor somatic firing and peak amplitude (Figure 6). Meanwhile, cp-AMPA current can effectively enhance somatic response and involve the sensitivity for dispersed synaptic input (Figure 7). The results on non-linear dendrites show that supralinear dendrites are more easily affected by synaptic conductance, and unlike changing the diameter, the firing property of the sublinear dendrites doesn't change. As dendritic spike initiation is associated with the rising rate of the somatic voltage response (Gasparini et al., 2004), and the local dendritic spike threshold becomes more depolarized as dV/dt decreases, we find that the specific morphology and cp-AMPA conductance will provide the required level of rapid dendritic depolarization (Figures 4B, 8B) (Golding and Spruston, 1998; Gasparini and Magee, 2006). These findings may support the essential role of increased cp-AMPAR transition induced by plasticity-related events in memory consolidation, retrieval, and updating (Torquatto et al., 2019).

Once action potential in GABAergic interneurons occurs will trigger GABA release (Martina et al., 2000). This process needs temporal and spatial integration precision. As for basket cells, they densely target the perisomatic region and will thus control the firing possibility of the pyramidal cell (Piskorowski and Chevaleyre, 2012). Therefore, how FS BCs spatially filter synaptic inputs is critical to their function in the operation of neuronal networks. Our work provides insight into the responses of FS BCs to spatial dispersion inputs, demonstrating the superiority of their specific morphology and cp-AMPA current on neuronal outputs. These results are likely necessary to generate precise signals for the temporal coding of information and control spike-timing-dependent plasticity at glutamatergic synapses.

## Data availability statement

The raw data supporting the conclusions of this article will be made available by the authors, without undue reservation.

## Author contributions

ML and XS designed the study, performed the research, analyzed data, and wrote the paper. All authors agree to accountable for the content of the work. All authors contributed to the article and approved the submitted version.
