# Supplementary figures and images for: Spatial integration of dendrites in fast-spiking basket cells
Source: Front Neurosci. 2023 Apr 4;17:1132980. doi: 10.3389/fnins.2023.1132980 (PMC10110864; doi:10.3389/fnins.2023.1132980)

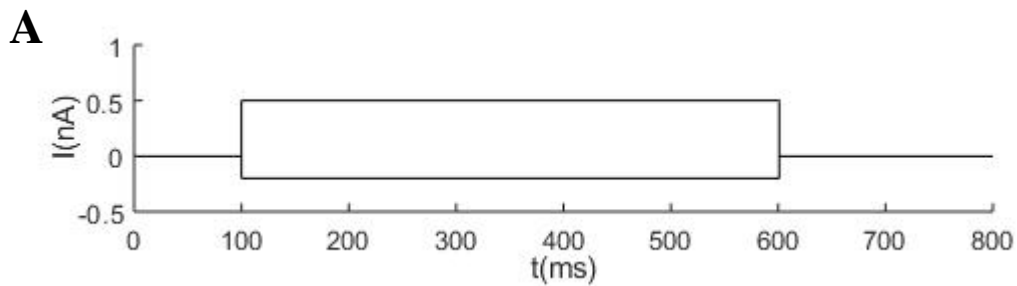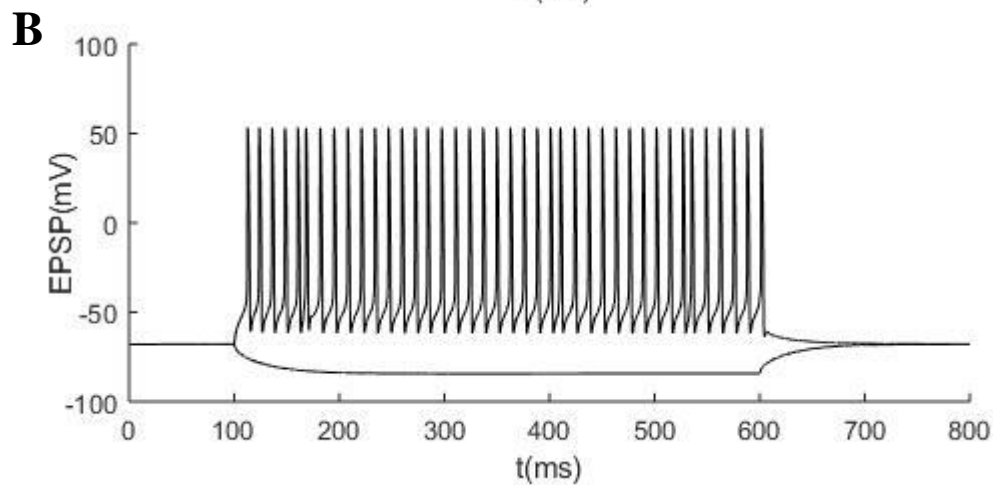

Supplement: Supplementary file 1 [file Data_Sheet_1.zip › frontiers_SupplementaryMaterial/9.pdf]
